# Immunoceptive inference: why are psychiatric disorders and immune responses intertwined?

**DOI:** 10.1007/s10539-021-09801-6

**Published:** 2021-04-30

**Authors:** Anjali Bhat, Thomas Parr, Maxwell Ramstead, Karl Friston

**Affiliations:** 1 Wellcome Centre for Human Neuroimaging, London, UK; 2 Division of Social and Transcultural Psychiatry, Department of Psychiatry, McGill University, Montreal, Canada; 3 Division of Psychiatry, University College London, London, UK; 4 Spatial Web Foundation, Los Angeles, CA USA

**Keywords:** Immunology, Psychiatry, Active inference, Immunoceptive inference, Autoimmunity, Diaschisis, Sensory attenuation, Pregnancy, Threat avoidance

## Abstract

There is a steadily growing literature on the role of the immune system in psychiatric disorders. So far, these advances have largely taken the form of correlations between specific aspects of inflammation (e.g. blood plasma levels of inflammatory markers, genetic mutations in immune pathways, viral or bacterial infection) with the development of neuropsychiatric conditions such as autism, bipolar disorder, schizophrenia and depression. A fundamental question remains open: *why* are psychiatric disorders and immune responses intertwined? To address this would require a step back from a historical mind–body dualism that has created such a dichotomy. We propose three contributions of active inference when addressing this question: *translation*,* unification*, and *simulation.* To illustrate these contributions, we consider the following questions. Is there an immunological analogue of sensory attenuation? Is there a common generative model that the brain and immune system jointly optimise? Can the immune response and psychiatric illness both be explained in terms of self-organising systems responding to threatening stimuli in their external environment, whether those stimuli happen to be pathogens, predators, or people? Does false inference at an immunological level alter the message passing at a psychological level (or vice versa) through a principled exchange between the two systems?

## Introduction

In recent years, evidence for the interconnection between psychiatric disorders and immune responses has been accumulating rapidly (Nutma et al. 2019). So far, these advances have largely taken the form of correlations between specific aspects of ‘peripheral’ immunity (e.g. blood plasma levels of inflammatory markers, genetic mutations in immune pathways, viral or bacterial infection) with the development of for neuropsychiatric conditions such as autism, bipolar disorder, schizophrenia and depression (Nudel et al. 2019). While these correlations speak to the interdependence of these two systems, there is less clarity in the literature as to why such a dependency should exist at all (Bennett and Molofsky 2019). This relationship is further confounded by the fact that the brain, which has been the primary physiological target of psychiatric research thus far (Oertel and Kircher 2010; David and Nicholson 2015), has some specialised immune characteristics (such as microglia, a cell species responsible for mediating immunity in the brain), and is physically sequestered behind the blood–brain barrier—licensing the common belief that the brain is ‘immune privileged’ (Bennett and Molofsky 2019). In essence, the question that remains unanswered and often unasked is, *why* are psychiatric disorders and immune responses intertwined?

To address this would require a step back from a dualism (Descartes 1641/1979), still subtly prevalent in modern medicine and contemporary philosophy (Putnam 1960, 1967; Morris 2010; Mehta 2011; Gendle 2016; Glannon 2020) between the mind (and often, in concordance, the brain) and the body. The complexity of the human brain, and its intimate relation to our conscious experience, makes it easy to forget that it is, nevertheless, an organ in service of maintaining the integrity of the body it inhabits. To reject this dualistic view is to view the mind as embodied, and the brain as a part of the living body (Varela et al. 1991).

The ripples of effect that pass between the brain and the immune system (Blalock 1984) are less surprising, however, under the hermeneutic perspective (Gadamer 1976; Friston and Frith 2015a, b) supplied by the free energy principle (FEP) (Friston 2005, 2009), in which autopoiesis—or self-evidencing (Clark 2013; Hohwy 2013)—is a constant process at every organismal level (cells, tissues, organs, organisms, societies), as well as a fundamental motivational drive. In this light, the brain and the immune system share a common imperative: to distinguish consistently and accurately between ‘self’ and ‘non-self’ or ‘threatening’ and ‘non-threatening’ to the individual as a whole. The multiscale perspective afforded by the free energy principle means this disambiguation between self and other is constrained by the hierarchical level (i.e. spatiotemporal scale) above (Kirchhoff 2018; Kirchhoff et al. 2018; Ramstead et al. 2018; Hesp et al. 2019; Ramstead et al. 2019; Palacios et al. 2020)—a necessary facet of ‘belonging to something greater’. On a general note, this thesis rejects dualism in the same spirit of recent proposals—from molecular biology (Kuchling et al. 2019; Manicka and Levin 2019) to evolution (Ao 2005; Frank 2012; Campbell 2016; Ramirez and Marshall 2017)—that put inference, beliefs^1^ and purpose into biological processes.

In this paper, we propose that an appeal to the FEP, and its corollary, active inference, is useful for explaining the relationship between the immune system and the brain in three important ways: *translation*,* unification*, and *simulation*. We will unpack this in five parts. In the first two sections, we briefly overview active inference and the human immune response. In the third, we explore insights that may be gained by translating the immune response into the language of active inference. In the fourth, we explain how understanding the brain and immune system as components of a larger Markov blanket explains their relationship in terms of a shared imperative and propose a ‘diaschisis of threat’ model that may elucidate the overlap between autoimmune and psychiatric disorders. In the fifth, we demonstrate the benefits of formulating these ideas in the form of generative models.

### Active inference and the Free Energy Principle

The Free Energy Principle (FEP) is a formalisation and extension of Schrödinger’s (1956) seminal observation that living organisms are defined by the avoidance of entropy—in other words, they ‘self-organise’, or maintain homeostasis. Supplied by the mathematics of nonequilibria, it emerges that all self-organising (and therefore biological) systems are fundamentally driven to minimise a quantity called ‘free energy’—which can be heuristically understood as a measure of unlikeliness.^2^

Active inference is an application of the FEP to sentient behaviour. It specifies that self-organising systems, in addition to adapting to their environment, can also *act* upon it so that it conforms to their internal, generative model of the world (Friston et al. 2010; Parr and Friston 2018, 2019). An internal model is a probabilistic account of how sensory data are generated—normally comprising a prior (how probable is a hypothesis before making any observations) and a likelihood (how likely are observed data under that hypothesis). For more sophisticated systems, this model may represent sequences through time, making it possible to select ‘policies’ (*sequences* of actions) that minimise ‘*expected* free energy’—which (heuristically) is the free energy expected on pursuing a policy. Some of these terms may seem somewhat anthropomorphic. This is because the origins of active inference were in application to the human brain, building upon Helmholtz’s (1866/1962) ideas about ‘unconscious inference’ and the concepts of the ‘Bayesian brain’ and ‘predictive coding’ (Rao and Ballard 1999; Knill and Pouget 2004)—equating free energy minimisation with ‘prediction error minimisation’, or ‘belief updating’.^3^

Under these frameworks, the internal dynamics of a biological system can be understood as solving an inference problem using sensory data. By combing prior beliefs with the likelihood associated with sensory data, we arrive at a posterior belief; namely, the probability of some explanation of observed sensory data. Behaviour is guided by these inferences (Friston et al. 2010; Adams et al. 2013a, b; Friston and Frith 2015a, b). Identifying the inference problem that the system is solving supplies an explanation, in the form of a generative model, that underwrites optimal behaviour*.* In a sense, this approach represents a formal rejection of Cartesian dualism in favour of a Markovian Monism (Friston et al. 2020). The first step in trying to understand the inference problem a system is implicitly solving is to define what is meant by ‘a system’. The statistical construct of a ‘Markov blanket’ (Pearl 1988) is typically applied to delimit a self-organising system, by rendering the internal components of the system conditionally independent from its environment, while accommodating a vicarious communication between the inside and the outside.^4^ This bidirectional communication is wrought by dividing the blanket into unidirectional influences that are either sensory (e.g. from pathogen to immune system) or active (e.g. from immune system to pathogen).

Further, under the Complete Class theorem (Wald 1947; Daunizeau et al. 2010), any behaviour can be rendered Bayes optimal given the appropriate prior beliefs. This means that defining the ‘inference problem’ can also help to explain (by lesioning the optimal generative model) maladaptive behaviours, such as might be seen in autoimmune or psychiatric disorders. This approach has been applied fruitfully to explain—for example—visual neglect (Parr and Friston 2018), hallucinations (Adams, Stephan et al. 2013a, b; Benrimoh, Parr et al. 2019) and failures of interpersonal communication (Moutoussis et al. 2014).

The implication for philosophy here is support from the physics of biology for a hermeneutic perspective (Gadamer 1976; Friston and Frith 2015a, b) of constant (and imperfect) energetic dialogue between an organism and its environment; and a relativism wherein normality is context dependent, perception is deeply subjective and absolute objective reality is unattainable.

### A primer on immunology

The human immune system is a sophisticated, multi-organ system that fights infection, prevents cancer, eliminates harmful substances, regulates inflammation and supports wound healing (Murphy et al. 2012; Portou et al. 2015; Marshall et al. 2018). It performs these functions by recognising tissue damage, differentiating ‘self’ from ‘nonself’, and destroying any foreign or toxic material. At the centre of this system are white blood cells, that move around the body through a network of delicate tubes and nodes, together called the lymphatic system (Murphy et al. 2012). On encountering disease-causing organisms, or pathogens*—*such as viruses, bacteria and parasites (Chaplin 2010; Murphy et al. 2012)—they enact an immune response. Two key types of white blood cells are macrophages (that engulf and dissolve pathogens and infected cells—a process known as phagocytosis); and lymphocytes, which further subdivide into B-cells and T-cells. T-helper cells (which are positive [ +] for the cell surface glycoprotein ‘cluster of differentiation’ 4, or CD4), release cytokine*s* (molecular ‘alarm’ bells that can initiate or attenuate an immune response); cytotoxic T-cells (with the surface marker CD8) can directly neutralise pathogens (Murphy et al. 2012). In health, these exist in a ratio of CD4 + to CD8 + T-cells of approximately 2:1 (McBride and Striker 2017). B-cells subdivide into plasma cells, which produce antibodies, and memory cells, which remember previously encountered antigens in case of future infections (Chaplin 2010; Murphy et al. 2012; Marshall et al. 2018).

#### Innate immunity

The innate component of the immune system mounts a relatively non-specific inflammatory response, which is tuned by the adaptive system. It comprises immune molecules and cells that detect, attack, and engulf pathogens. A useful starting point in understanding this system is the complement pathway: a series of ‘molecular dominoes’ that trigger a cascade of events designed to neutralise any pathogens. The molecules that comprise complement system are plasma proteins known as *complement components*, denoted as ‘C1, ‘C2′, ‘C3′, and so on (Murphy et al. 2012). Each of these has a unique role, as outlined below.

There are three ways in which this cascade may be triggered (Chaplin 2010). The first is known as the *classical* pathway, and rests upon binding of complement component C1q to IgG or IgM antibodies.^5^ The implication here is that, in the presence of a pathogen identified by the adaptive arm of the immune system, there will be a high density of antibodies to which C1q may bind.^6^ This leads to a localised increase in activity of the classical pathway. The second complement pathway is the *alternative* pathway, which is tonically active—possibly embodying a belief about the prior probability of infection. The third is the *lecithin* pathway. Like the classical pathway, the lecithin pathway is triggered by the binding of endogenous molecules (mannose binding lecithin) to antigens (mannose) on the surface of pathogens. Crucially, this does not require the production of antibodies by the adaptive immune system. One interpretation of this pathway is in signalling the likelihood of infection. The presence of mannose indicates a high likelihood, while its absence indicates a low likelihood.

These three pathways converge upon the C3 convertase enzyme, which breaks C3 down into C3a and C3b. C3a sets in motion events that facilitate immune cells entering the tissues from the blood. It does so through triggering degranulation of mast cells in the tissues.^7^ These release histamine that acts to increase vascular permeability (Ashina et al. 2015). C3b inhibits further action of C3 convertase, while additionally triggering the breakdown of C5 into C5a and C5b. C5a acts as a chemoattractant for circulating neutrophils, which pass through the permeable vasculature into the tissues. C5b joins forces with C6, C7, C8, and C9 to form the membrane attack complex, which is used to punch holes in the surface of the pathogen. The neutrophils (and tissue macrophages) engulf the pathogen through a process known as phagocytosis and produce reactive oxygen species to kill these pathogens.^8^ Tissue macrophages may respond to pathogens independently of the complement pathway as they (like C1q) can sense the presence of Fc regions on IgG and IgM antibodies (Chaplin 2010; Murphy et al. 2012). Foreshadowing some of Sect. 3, we could interpret this as an example of an action-perception cycle, where increased C3 convertase activity corresponds to a primitive kind of percept, whose (active) consequences are the neutralisation of pathogen.

#### Adaptive immunity

There are several points at which the adaptive arm of the immune system tunes this response. It does so by producing antibodies (also known as ‘immunoglobulins’), which are Y-shaped proteins produced by plasma cells. Each tip of the ‘Y’ has a binding site with a unique structure, allowing each antibody to bind with high specificity to ‘antigens’, which are unique molecules on the surface of (or released by) cells and microorganisms. The specificity of this binding acts as a ‘lock-and-key’ mechanism that can identify antigens, and mark known pathogenic or unknown antigens for destruction by macrophages. Once pathogens are coated with antibody, all the events outlined in the previous subsection are initiated. The presence of specific antibodies favour increased classical complement pathway activation (perhaps acting as an ‘empirical’ prior for this system^9^), increased degranulation of mast cells, and increased phagocytosis by tissue macrophages in response to specific antigens (Murphy et al. 2012).

A good place to start in reviewing this system is the Major Histocompatibility Complex (MHC), also known in humans as the Human Leukocyte Antigen (HLA).^10^ MHC comes in two flavours (I and II). MHC-I is found on the surface of almost all somatic cells (Murphy et al. 2012). Once a cell has become infected by an intracellular pathogen, it uses the MHC-I to display antigens from that pathogen on its surface. MHC-II is used similarly but is only present on the surface of specialised immune cells that engulf and phagocytose pathogens (Chaplin 2010). These include macrophages, B-cells, and dendritic cells—collectively known as antigen presenting cells (APCs).

The MHC-I pathway allows an arm of the adaptive immune system to interact directly with pathogens, without needing to go through the innate immune system. These mechanisms occur in peripheral tissues and circulation. T-cells with surface CD8 receptors bind to the MHC-I and, if the antigen presented by this molecule matches the specificity of that cell’s T-cell receptor (TCR), the CD8 + T-cell releases perforin, granulysin and granzyme, which trigger the death of the infected cell.

The MHC-II system sits a level above the innate and MHC-I systems (Zhang et al. 2014). Once the innate immune system has enabled various APCs to engulf pathogens and display their antigens via MHC-II, these cells travel to lymph nodes where they are met by CD4 + T-cells. Like CD8 + cells, these have antigen-specific TCRs that bind to MHC-antigen complexes but are selective for MHC-II. On binding, CD4 + T-cells differentiate into Th1 or Th2 cells, depending upon whether they are dealing with an intracellular or extracellular pathogen, respectively.

Th1 cells release interferon-γ (IFNγ) that triggers macrophages to destroy any pathogens they have engulfed. In addition, they induce antibody production by B-cells. Th2-cells recruit Eosinophils^11^ through interleukin (IL) 5 secretion, and promote isotope switching in B-cells through IL-4 signalling. Heuristically, the Th1 effect over B-cells is to increase specific antibody production. The Th2 effect is to broaden the distribution of antibody specificities (Murphy et al. 2012).

The process of B-cell activation by a Th1-cell occurs in lymphoid tissue. B-cells in the periphery bind to a pathogen via their B-cell receptor (a membrane bound antibody) and endocytose it. As outlined above, they present antigens to Th1-cells via MHC-II. On binding of the TCR to MHC-II, the T-cell presents a CD40L molecule that binds to the B-cell CD40 surface molecule (Elgueta et al. 2009). This stimulates the B-cell to differentiate into either a plasma cell (secreting antibodies) or a memory cell (a simple form of immunological plasticity). Although T-cell independent B-cell activation is a well-recognised phenomenon, this is outside the scope of this paper. The presence of antibodies towards a specific antigen effectively orients the complement system (via the classical pathway) to respond with greater amplitude to that antigen (Chaplin 2010). An analogy in cognitive sciences might be attentional orientation towards a visual stimulus, directed by descending messages from higher to lower cortical regions (Büchel et al. 1998; Buschman and Miller 2007). For more detailed overviews of the immune response, please see Marshall et al. (2018) and Murphy et al. (2012)

### Translation

Although the primary focus of the active inference literature so far has been the human nervous system, the immune system is a similarly complex dynamic system that may be explained using the same mechanics (Parr et al. 2020). In this section, we first present an example of translation of the immune response, as described above, into the language of active inference. We then present an example of what this may lend to the study of immunology.

#### The Markov blanket

As mentioned above, the first step in identifying the inference problem a system is solving is defining the limits of the system (i.e. the Markov blanket) and its active and sensory components. In the (simplified) immune response we describe here, the innate immune system ‘senses’ extracellular pathogens through Fc-regions of antibody (IgG or IgM) bound to specific antigens, binding of IgE antibodies on the surface of mast cells to pathogenic antigens, or through detection of cell-surface molecules such as mannose by mannose-Binding lecithin. Intracellular pathogens are sensed via binding of T-cell receptors to cell surface Major Histocompatibility Complex (MHC). For most cells, this is MHC I. For antigen presenting cells (including macrophages, dendritic cells, and B-cells), MHC II may also be used. This suggests at least three sorts of sensory influences:

1. Mannose-binding lecithin
2. Specific antigens
3. MHC-I

These sensory data are generated by external states comprising the specific pathogen, the presence of mannose on the surface of the pathogen, and whether a pathogen is intracellular or extracellular. There are many other molecules and sensors that play a role in detection of pathogens, but we focus upon the above three.

Active influences on these external states include release of molecules by CD8 + T-cells that lead to death of cells with intracellular pathogens. This leads to a decrease in antigen-presenting MHC-I sensory influences. In addition, the membrane attack complex from the complement pathway acts to kill pathogens in a relatively non-specific way, depleting both specific antigens and local concentrations of mannose-binding lecithin. Finally, extracellular pathogens are depleted by the action of macrophages and neutrophils that engulf these cells.

The three active influences we will focus upon are:

1. CD8 + T-cell molecules (perforin, etc.)
2. Membrane attack complex
3. Macrophages and neutrophils (phagocytosis)

The interactions between the three sensory and active influences we have identified may be thought of as analogous to spinal and brainstem reflexes of the sort found in the proprioceptive branches of the nervous system. Changes in the sensory aspect induce changes in the active part that restores sensations to some set-point. In the nervous system, the set-point depends upon descending signals from the brain that may be thought of as predictions (Adams et al. 2013a, b) of the proprioceptive consequences of the desired (i.e., anticipated) movement. In the field of motor control, this is known as the equilibrium point hypothesis (Feldman and Levin 2009).

#### The generative model

**Fig. 1:**
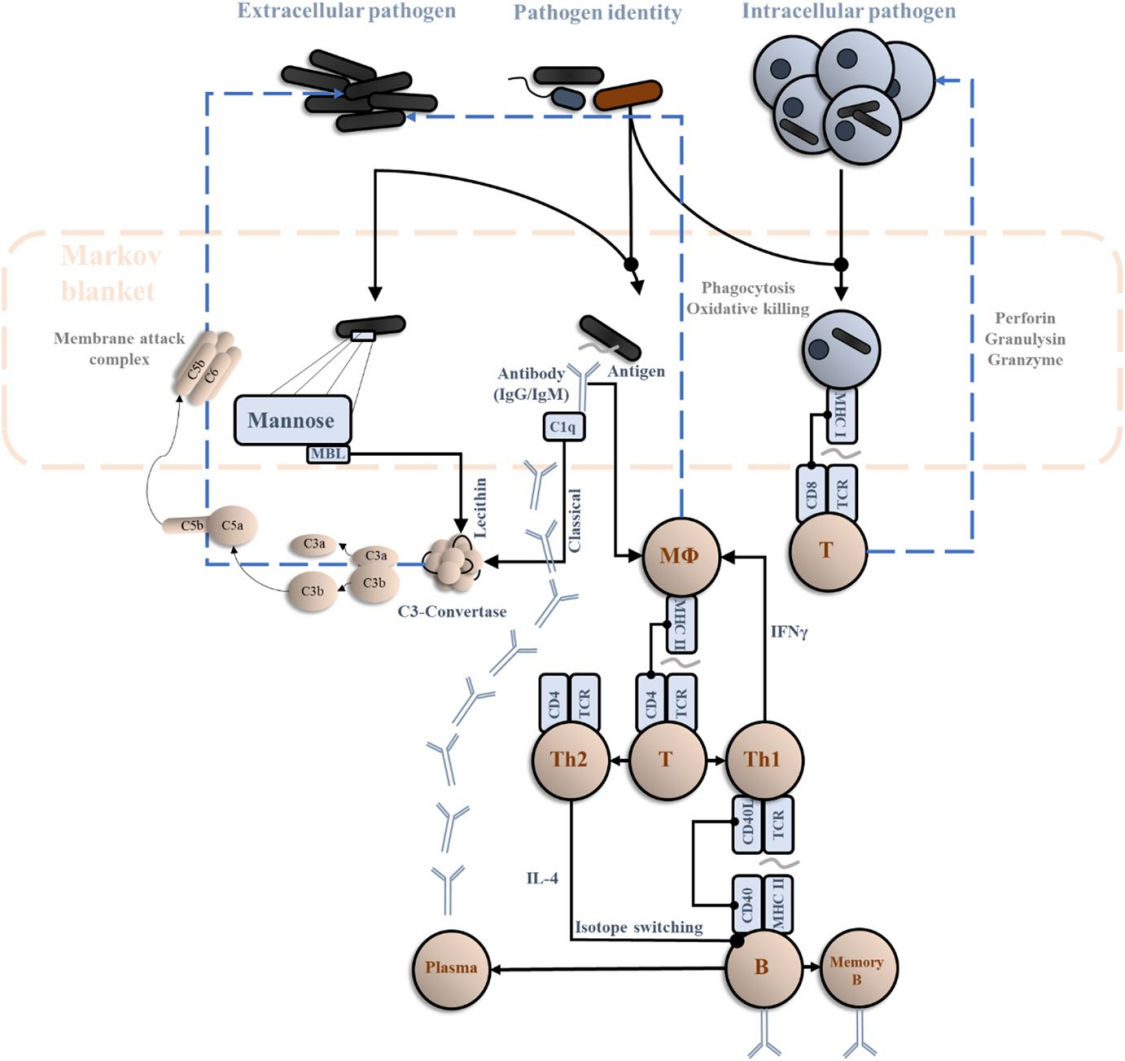
*Immunoceptive inference.* This schematic shows a simplified account of an immune response to foreign pathogens. It is arranged in the style of graphics used in computational neuroscience—and machine learning—to show a data-generating process comprising external states (here, the identity of the pathogen, and the intracellular and extracellular pathogen concentrations), the sensory data they generate (mannose, specific antigens, and MHC-I antigen presentation), and the message passing in the internal system—whose role is to draw inferences about external states and select actions that correct deviations from a desired state. The dashed blue lines emphasise the influence the internal states exert on external states (via active states). Note the resemblance between these and the simple reflex loops associated with movement generation in the spinal cord. The implication of this graphic is we can think of the internal states that influence these responses as like the neurons of the central nervous system, forming inferences about the outside world through message passing among different populations. One perspective on this is that the concentration of CD8 + T-cells represents an implicit belief about the number of infected cells, the concentration of macrophages (and activated C3-convertase) a belief about the number of extracellular pathogens, and the CD4 + T-cell to B-cell to plasma cell loop an example of message passing to identify the pathogen identity to direct ‘attention’ towards the appropriate antigens

Once we have defined the active and sensory states of the system, the challenge is to find the generative model that accounts for the dynamics of internal and active states.^12^ The model should specify which explanatory variables (external states) conspire to generate the sensory states. As shown in Fig. 1, the entirety of the second section of this manuscript can effectively be condensed into a single model and its inversion. Note that Markov blanket is an informational separation from the environment—it does not necessarily correspond to physically materialised boundaries (Kirchhoff et al. 2018; Palacios et al. 2020). The Markov blanket shown below is not comprised of a cell or tissue membrane but elements of the immune system (e.g. perforin molecules, macrophagic cells) that mediate the interactions with the pathogen. From this perspective, everything shown above the Markov blanket in Fig. 1 is the set of external states that generate the sensory states shown within the blanket. The dynamics of internal states (depicted below the blanket) can then be interpreted as drawing inferences about the external states, which then influence the active states in the Markov blanket.

#### Sensory attenuation in the immune system

If indeed active inference is a universal framework across self-organising systems, it stands to reason that key aspects of brain-based sentience explained by active inference may possess analogues in the immune system. We hinted at some of these analogues in Sect. 2 but unpack this in greater depth here. For example, the phenomenon of sensory attenuation (Brown et al. 2013) has drawn upon the notion, under active inference, that a system cannot act without temporarily attenuating the precision (gain) of the consequences of its own actions. This is because attenuating sensory precision effectively allows the system to ‘ignore’ the prevalent sensory evidence that “I am not acting”, thereby permitting a posterior commitment to the prior prediction, “I am acting”. These predictions are fulfilled by motor, autonomic or possibly immunological reflexes to realise the predicted sensory state of affairs. It is therefore action that, ultimately, updates the internal model, through an exchange with the external world (Friston and Frith 2015a, b).

If sensory attenuation possesses an immunological analogue, there may be a great deal to be learned by translating what has already been well-studied in the domain of neuroscience to the domain of immunology. If this is not the case, there is another, equally interesting avenue to be explored, in the form of the question, “What is different about the nervous system that makes its actions dependent upon sensory attenuation, when the actions of other physiological systems are not?” In addition, this kind of translation may also serve as a sanity check of sorts for claims made under the active inference framework.

To exemplify this, let us propose a plausible immune analogue. There will generally be an immune response triggered by the proliferation of allogenic cells and tissue damage in the body. However, there are some notable instances, such as pregnancy, when the body must tolerate the proliferation of allogenic cells and some degree of tissue damage, up to a certain threshold—at which labour is initiated. In order to allow a foetus to grow, it could be said that there must be an attenuation of the ‘sensory’ consequences (e.g. MHC-I presentation, which initiates an immune response) of self-generated proliferation. Indeed, foetal tissue is one of the few somatic tissues whose cells exhibit significantly reduced MHC-I presentation (Gaunt and Ramin 2001). Given the model above, it would be possible to start to explore this possibility by, for example, reducing MHC-I presentation as a sensory state. We intend to expand on this angle in future work.

The usefulness of drawing such an analogy is in effectively ‘stealing’ dynamic characteristics of Markovian systems from previous work. We are still in very early stages of understanding the profound immunological consequences of pregnancy. Sensory attenuation has been relatively well studied in the nervous system, and there may be significant insights to borrow from this literature. For example, previous simulations and experimental work (Parees et al. 2014; Limanowski et al. 2018) have shown that a failure of sensory attenuation can lead to pathological alterations in self-generated actions—for example, the deficits in motor control seen in Parkinson’s disease. In the immune analogue of pregnancy, a failure of ‘sensory’ attenuation could result in miscarriage or pre-eclampsia (Laresgoiti-Servitje et al. 2010). This could again be explored using a generative model (and its inversion) similar to that in Fig. 1 by, for example, adjusting parameters of the prior or likelihood such that the concentration of Th1 cells (which produce pro-inflammatory molecules)^13^ declines (or fails to do so). One manipulation that might achieve this is to attenuate the precision of the (likelihood) mapping from pathogens to MHC-I antigen presentation. Under the belief that the latter is not necessarily a consequence of the former (a valid belief in the context of pregnancy), we would expect a smaller update in beliefs about pathogens on observing MHC-I antigen presentation. If Th1 cell concentration embodies some aspect of this belief, this implies a smaller increase in this population of cells in response to MHC-I.

### Unification

#### Neuroendocrine regulation of immunity

The above outlines insights that may be gained by applying theoretical neurobiological methods to the functioning of the innate and adaptive immune systems. However, our primary interest is in the interface between these systems and the brain. Elements of this interface are direct, but much of the interaction is via the hypothalamic–pituitary–adrenal (HPA) axis. Briefly, the hypothalamus synthesises corticotrophin-releasing hormone (CRH) that stimulates the pituitary gland to release adrenocorticotrophic hormone (ACTH). This acts upon the adrenal gland to stimulate cortisol release. In addition to suppressing further ACTH and CRH release, cortisol suppresses activity of Th1-cells and macrophages. In fact, corticosteroids are frequently used in clinical practice to suppress inflammation (Cole and Schumacher 2005, Gegel et al. 2019). In turn IL-1, IL-6, and tumour necrosis factor (TNF) released by these cells normally increase hypothalamic release of CRH. Interestingly, CRH receptors are also found in the hippocampus, amygdala, and locus coeruleus (Herman et al. 2016).

**Fig. 2:**
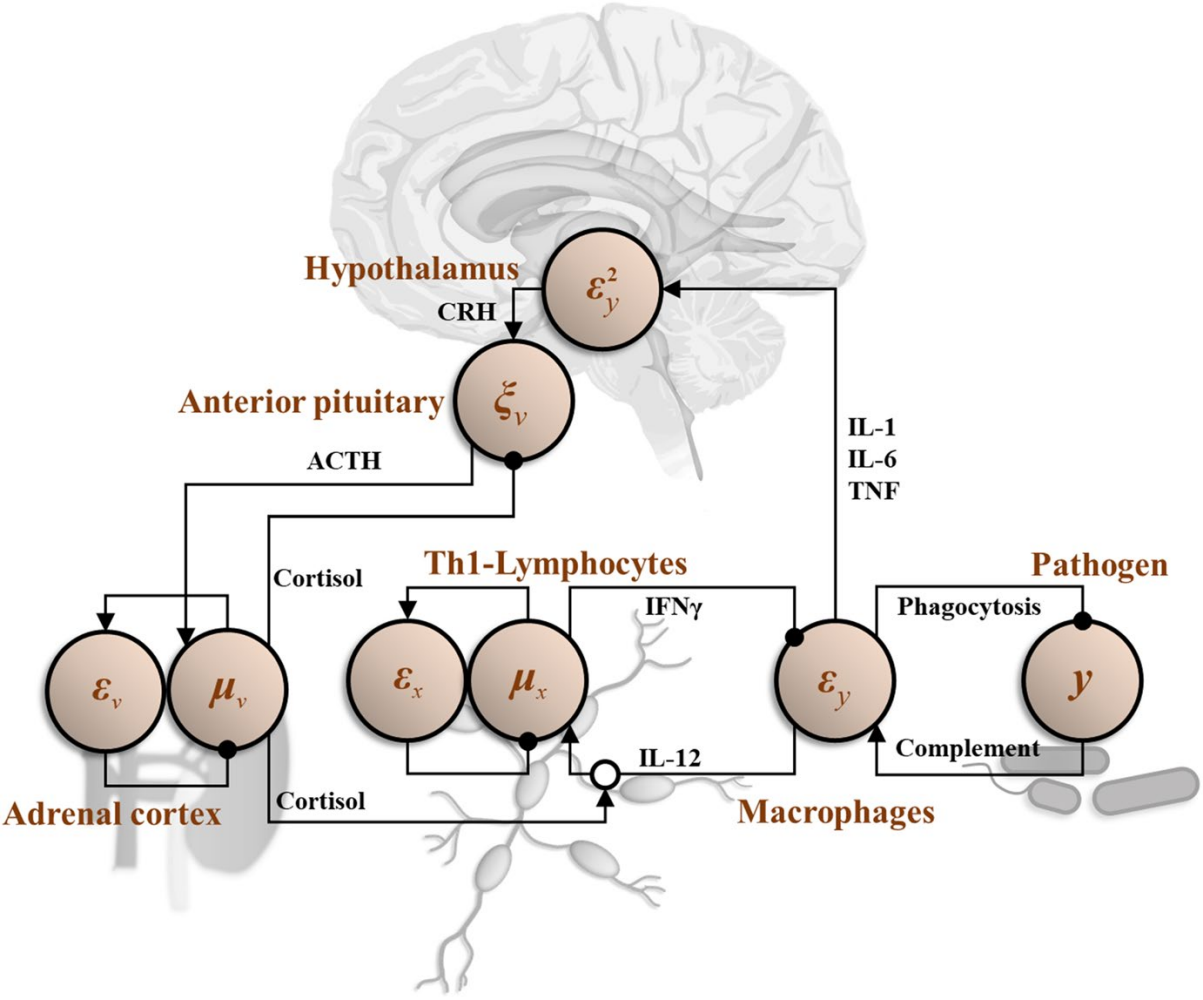
*Neuroendocrine regulation of immunity* via *the Hypothalamic–Pituitary–Adrenal axis.* In this graphic, we show the predicted pathogen concentration signalled by IFNγ derived from Th1-lymphocytes. This is subtracted from the complement pathway activation (playing the role of sensory data) detected by macrophages to give a prediction error (*ε*_*y*_) represented by the IL-12 levels released by macrophages. Intuitively, the presence of unanticipated pathogens prompts an increase in macrophage activation. This prediction error may be resolved in two ways. The first is to decrease the amount of pathogen through phagocytosis and oxidative killing. The second is to increase the Th1 response (*μ*_*x*_) to update predictions (IFNγ) so that they are consistent with the presence of pathogen. The degree to which the Th1 response is increased depends upon two things. The first is prior beliefs about the amount of pathogen expected. Deviation from this prior is indicated by the prediction error (*ε*_*x*_), which may be intrinsic to T-cell populations. The second is the precision or inverse variance associated with the predicted pathogen concentration. If the variance is assumed to be very high, the effect of the prediction error (*ε*_*y*_) on the expectation (*μ*_*x*_) is attenuated. Here, we have assumed the expected variance is a function of some variable *v* whose expectation (*μ*_*v*_) is signalled by cortisol from the adrenal cortex. This means that, when cortisol is high, the Th1 response to macrophage-derived cytokines is more limited. To update beliefs about variance, we can penalise deviations from a prior value as before (*ε*_*v*_), but the prediction error from *y* has to be handled more carefully. As variance is a second order statistic (the expectation of a squared quantity), we need to square the prediction error (as shown in the hypothalamus) and compare this to the current estimate of the variance. These (respectively) account for the cytokines released by macrophages and detected by the hypothalamus, and for the negative feedback from the adrenal cortex to the anterior pituitary—shown as the point at which the square prediction error and variance are compared (with *ξ*_*v*_ representing their ratio)

In addition to the HPA axis, the hypothalamus directs immune responses through the autonomic nervous system. The sympathetic branch of this innervates lymph nodes directly (Kenney and Ganta 2014). The hypothalamus also directly orchestrates the fever response to infection. As such, the hypothalamus may be seen as an interface between the immune system and the central nervous system. The importance of this role has been demonstrated in empirical studies (Alaniz et al. 1999; Barrios-Payán et al. 2016) and has been central to developments in theoretical immunology (Rosas-Ballina and Tracey 2009; Tracey 2009). This is important because, if there are physiological interfaces between the immune system and the brain, then these systems can be understood as jointly optimising a shared generative model (i.e. a Markov blanket can be drawn around both of them). Figure 2 depicts the HPA axis as a (simplified) example of the message passing that might emerge from inversion of a shared generative model between the immune system and the brain.

#### Neuroimmunological diaschisis

Typically, the interaction between the brain and the immune system is studied by treating the two as separate systems and asking how the immune system might attack the nervous system. The advantage of framing the nervous and immune systems as a *single* system—that solves a single generative model—is that it offers the opportunity to think about a neuroimmunological ‘diaschisis’. A diaschisis (literally, ‘shocked throughout’) is a functional change in distant parts of a system following a localised lesion (Price et al. 2001; Finger et al. 2004; Carrera and Tononi 2014; Fornito et al. 2015). The classical example of this is hypometabolism of the contralateral cerebellum following a motor-cortical lesion (von Monakow 1914).

As an example of such a shared generative model, Fig. 2 presents an interpretation of the neuroendocrine interface with the immune system in terms of a predictive-coding style message passing architecture. This is the sort of message passing that arises from writing down a specific kind of generative model. The implicit model in question here is inspired by models used to account for precision estimation (Kanai et al. 2015). Intuitively, we can think of this as prediction of some observable (from the perspective of the immune system) characteristic of a pathogenic population (e.g., its concentration), represented by the variable *y*. This prediction has two parts: (1) the expected concentration of that population (given by *μ*_*x*_) and (2) the variance expected around that expectation [given by exp(*μ*_*v*_)]. The implication is that we can account for the HPA axis and its relationship with the immune system by assuming a generative model in which Th1-lymphocytes predict the concentration of some pathogen, and cortisol represents a prediction about the uncertainty of that prediction. The negative feedback loops, characteristic of these systems, then emerge from the message passing used to update these Bayesian beliefs.

The idea here is that abnormal neural computation could arise from an immune lesion, because the (otherwise healthy) signalling from immune cells to neural tissue is altered. Similarly, psychiatric or neurological insults might lead to abnormal neural regulation of immunity. We can see how this could work in Fig. 2, noting the presence of CRH receptors in multiple brain regions. A polymorphism in a receptor in the immune system (e.g., the Th1 IL-12 receptor) might lead to changes in the release of cytokines by macrophages, changing the values of the variables represented in the hypothalamus. This changes the information available to other parts of the brain that respond to CRH. Note that this does not involve the immune system attacking the nervous system—the latter may respond optimally based upon the information available to it.

#### A diaschisis of vigilance?

Learning to appropriately infer threat is an essential and highly conserved facet of biological systems (Bach et al. 2018; Ojala and Bach 2020). It is of great importance that these inferences be accurate.^14^ Too much avoidance (or hypersensitivity) excessively and unnecessarily limits the interactions between the system and its environment, effectively starving it of (epistemic) resources; too little avoidance (hyposensitivity, or naïveté) can unnecessarily expose the system to risk. The brain and the immune system can certainly be seen as engaged in avoiding threats to their own integrity and that of the organism as a whole.

‘Hypersensitivity’ is a usefully intuitive term here, as it generalises well. Disproportionate and misdirected activity of the immune system is often a result of disorders collectively called hypersensitivities. These include allergies and autoimmune disorders, when the system mistakenly perceives its own tissues as threatening. Such conditions may result from, for example, variation of genes related to immunity, or environmental sensitisation. A number of central symptoms of psychiatric disorders can also be understood as hypersensitivities—such as social threat hypersensitivity in borderline personality disorder and depression (Bertsch et al. 2013; Slavich and Irwin 2014; Badcock et al. 2017) or sensory hypersensitivities in autism (Takarae et al. 2016). There are several well-established links between hypersensitivities and psychiatric disorders; for example, systemic lupus erythematosus (SLE) and depression (Moustafa et al. 2020); thyroiditis and anxiety (Siegmann et al. 2018); maternal diabetes type 1 and autism (Xiang et al. 2018); SLE, psoriasis, rheumatoid arthritis and schizophrenia (Tiosano et al. 2017; Chen et al. 2019; Ungprasert et al. 2019). Indeed, some accounts even suggest that schizophrenia *is* an autoimmune disorder (Knight et al. 1992; Adams et al. 2012).

Through the lens of neuroimmunological diaschisis, an interesting question may be raised here. Under the hierarchical perspective of active inference, the brain and the immune system are internal states of the same Markov blanket and necessarily influence each other (Kirchhoff et al. 2018, Palacios et al. 2020). If one process (e.g. the immune response) within a larger Markov blanket is faced with a threat to its integrity, are other processes (e.g. psychological aversion) within that blanket primed towards threat avoidance as a result? If this is the case, an important story could be told about how and why immune insults—especially early in life or in utero—are linked to the manifestation of psychiatric disorders even decades later (Guma et al. 2019), and why people with certain psychiatric disorders are more likely to have allergies, autoimmune conditions, and to suffer from other hypersensitivities (Benros et al. 2011; Benros et al. 2013; Benros et al. 2014).

Computationally, for this to be true, there must be a possibility of generalisation of prior beliefs about threat (and their precisions), both between concepts and across physiological systems within a Markov blanket. There is evidence from theoretical and behavioural work demonstrating the generalisation of prior beliefs and precisions across conditions (Kawashima and Kusnecov 2002; Fernandes et al. 2014). We plan to expand on this notion in future work by considering whether there is an optimum degree of generalisation of threat avoidance between physiological systems.

A well-established model of threat learning in mammals is Pavlovian fear conditioning (Bach et al. 2018; Ojala and Bach 2020), in which a neutral (‘conditioned’) stimulus is paired with a threatening (‘unconditioned’) stimulus such that an association is developed between the two. The result is that the neutral stimulus eventually engenders an aversive response even without the presence of the unconditioned stimulus. Experiments that lesion threat memory are challenging to conduct in human populations. In the next section, to illustrate the benefits of taking a theoretical approach, we outline an example of an in silico experiment that offers the opportunity to explore the effects of lesioning threat memory.

### Simulation

Wet lab-based work that usually advances immunology is often expensive and time-consuming and clinical studies of immune and neurological disorders are usually faced with ethical restrictions. A major advantage of this kind of theoretical approach is in providing a proof principle that validates the various costs of pursuing a new hypothesis empirically. Translated into a generative model, an experiment can be simulated in silico with the requisite flexibility to define specific experimental and environmental parameters, which generate data. Or, trained on existing empirical data, it is possible to generate sophisticated predictions about outcomes given new data. For example, in recent work, we have used a similar modelling approach to investigate susceptibility to symptoms of, and likelihood of testing positive for, Covid-19 (Parr et al. 2020).

While this (conceptual) paper is not the place for introducing new mathematical models or simulations, it is useful to think about how we would construct a generative model from which simulations could be developed. A challenge often faced by computational biology is the combinatorial complexity that cannot but be simplified for the purposes of simulation: biology is as messy as physics is neat. The advantage of the active inference approach is that if we can define the problem the system is solving, the Bayes optimal solution to this problem automatically tells us what the relevant (internal state) dynamics are. This lets us take a more focused, teleological and ‘top-down’ approach to understanding the neuroimmunological system, as opposed to trying to build up a model by writing down the dynamics of each component of the system and hoping for an emergent pattern.

In neurobiology, we typically start by selecting an experimental paradigm that involves presenting participants with some problem (sensory discrimination, decision-making, etc.) that we know the brain can solve. To be able to solve such problems implies the brain’s model of the world accurately accounts for how we (as experimenters) have generated the stimuli that were presented to the participants. Formalising this and computing the optimal solution tells us about the structure of that solution. This typically involves a network of beliefs, with messages passed along the links of that network like action potentials along axons in a brain. This means we do not need to attempt to model the entire brain, and instead can focus upon the minimal networks required to explain the phenomena of interest.

Here, we consider the same construct, applied to networks that include message passing among elements of the immune system. The key challenge here is to identify the right sort of experimental paradigm, and to think about how that might be represented as a generative model. We illustrate the principles of this in relation to an existing experiment that demonstrates a neuroimmunological diaschisis. This is based upon a taste-aversion classical (Pavlovian) conditioning paradigm (Ader and Cohen 1975, 1991), in which rats were first injected with an immunosuppressant called cyclophosphamide (the unconditioned stimulus), or a placebo, and simultaneously fed either a saccharin-flavoured drinking solution (the conditioned stimulus) or plain water. This meant there were three groups. Group 1 were given saccharin and cyclophosphamide, group 2 were given plain water and cyclophosphamide, and group 3 were given plain water and a placebo. They were then injected with sheep red blood cells (i.e. foreign material that would typically induce an immune response). Three days later, some of the mice were re-exposed to saccharin. Ader and Cohen (1991) found that conditioned (cyclophosphamide-treated) rats showed a heightened aversion to saccharin (intuitively, this is similar to the human experience of an acquired aversion to foods consumed just before a period of illness); as well as, interestingly, a reduced immune response to sheep blood cells compared to placebo-treated rats and treated rats not re-exposed to saccharin.

**Fig. 3:**
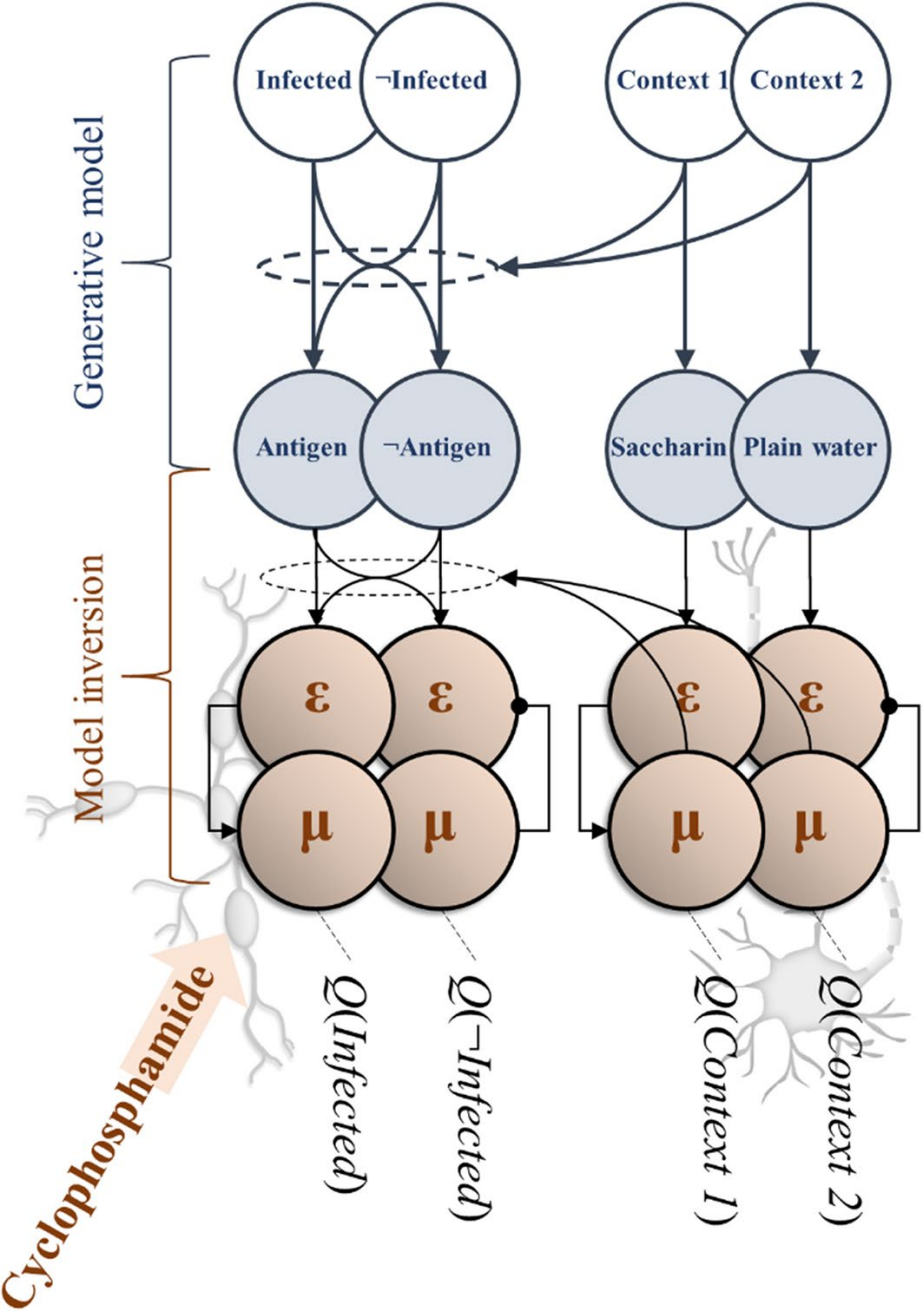
*Classical conditioning of the immune system.* This figure is a graphical representation of the classic taste aversion experiment by Ader and Cohen (1991). Here, the unfilled circles at the top represent the hidden states of the generative model (the saccharine-context for the nervous system and the presence of infection for the immune system). The filled blue circles represent sensory states or ‘observations’ (e.g. MHC-I presentation of sheep red blood cell antigens). The ε and μ symbols represent prediction errors and expectations (of a categorical sort) of the nervous (right) and immune (left) systems, which encode probabilistic beliefs (*Q*) about the hidden states. Cyclophosphamide treatment (unconditioned stimulus) suppresses the immune response, which precludes an ‘infected’ inference in the presence of antigen. We could interpret this as attenuating the precision with which antigens are predicted (allowing for some probability of detection in the non-infected condition, and for some probability of non-detection in the infected condition). If this happens in the presence of saccharin only, this attenuated precision may be learned in a context specific way. Eventually, the presence of saccharin (conditioned stimulus), leading to an inference of ‘context 1’ implies low precision in the immune modality, and an attenuated immune response even in the presence of antigen

In Fig. 3, we illustrate the way in which this experimental design could be represented as a generative model. In addition, it shows the message passing scheme which could invert a model of this sort. The key features are the division into two streams (left and right) that deal with inferences about whether or not ‘I am infected’, and about the (gustatory) context. The former relies upon the (immunoceptive) detection of antigens, while the latter relies upon the presence or absence of saccharin (involving the central nervous system). Despite this division between the two streams, this scheme models neuronal modulation of gain (precision) of the immune response. Classical conditioning can thus be understood as the process of learning about the temporal and/or causal relationships between external and internal stimuli. The value of formulating a model in this way is threefold. First, as alluded to above, it lets us select the minimal set of nodes in a message passing scheme that we need to be able to explain some facet of behaviour in an otherwise very complex system. Second, it gives us some intuition as to what the neuroimmunological system is ‘trying to do’, in the sense that the dynamics are now seen as solving an inference problem. Finally, it is consistent with the kinds of formulation used in computational neuroscience, enabling development of simulations for synthetic experimentation.

## Conclusions

In this paper, we have introduced ‘immunoceptive inference’: active inference from the perspective of the immune system. This is in a similar vein to the notion of ‘interoceptive inference’, which frames emotions as emerging from—or perhaps furnishing—predictions about the causes of visceral sensations. In brief, interoceptive inference claims the brain is continuously updating predictions about, and acting upon, the body it inhabits (Seth 2013). In our formulation, the body itself (in this case, the immune system) is seen as furnishing predictions of—and acting upon—sensory input, informing ‘beliefs’ about whether an antigen belongs to the category of ‘self’ or ‘nonself’.

In so doing, we have highlighted three practical contributions (translation, unification and simulation) of the active inference framework to answering and—crucially—redefining the question, “Why are psychiatric disorders and immune responses intertwined?” We suggested that it is inevitable that two systems within the same Markov blanket influence each other: the brain and the body together make predictions about exteroceptive, interoceptive, and immunoceptive input. To this end, we have proposed an example of a common generative model that the brain and immune system jointly optimise, treating molecular components of the immune system as sensory or active states and the resulting cellular response as message passing at lower levels of a ‘sensory’ hierarchy that interfaces with the brain. Our scheme expresses the classical conditioning of the immune system in terms of inference at an immunological level, that may alter the message passing at a psychological level (or vice versa) through an optimal interface between the two systems.

This surrender of mind–body and brain-body dualisms may be of particular importance to psychiatric practice, where it encourages a holistic treatment of patients. For example, with an embodied perspective on the mind, a patient presenting with psychosis may be treated with reference to the mechanisms leading to this syndromic endpoint, whether that be schizophrenia (treated with antipsychotics), or an alternative (e.g., endocrine) diagnosis such as Cushing’s syndrome, which can be effectively treated by normalising cortisol levels (Tang, O'Sullivan et al. 2013, Wu, Chen et al. 2016)—or indeed autoimmune encephalitis (Symmonds et al. 2018). We also advance the possibility of drawing immunological analogues of concepts defined under active inference for neurological phenomena, such as sensory attenuation. Finally, we introduce the novel concept of neuroimmunological diaschisis and the possibility of a diaschisis of threat-avoidance that may contribute to the overlap between psychiatric disorders and immunological hypersensitivities. This kind of overlap leads to clear empirical predictions; for example, an association between psychopathology and (measurable) immunological responses, much in the same way that clinical tools such as the dexamethasone suppression test leverages the link between neuroendocrine function and stress or depression (Naughton et al. 2014).

## Footnotes

1 In this article, ‘beliefs’ should be read as Bayesian beliefs—in the sense of Bayesian belief updating and belief propagation. In other words, beliefs are simply posterior or conditional probability distributions, usually encoded by the physical state of a person or particle. They are not propositional beliefs of a pre-theoretical sort.

2 Strictly speaking, variational free energy is more generally an upper bound on surprisal, a.k.a. self-information; namely, the log probability that any given person or particle will be found in a particular state. In Bayesian statistics, negative self-information is also known as log evidence. Therefore, minimising free energy maximises evidence; hence, self-evidencing.

3 The updating of the generative model depends on a weighing up of the relative ‘precision’ of prior beliefs and sensory evidence, similarly to how a scientist would weigh up new evidence against a body of literature. Here, ‘precision’ is a measure of certainty, thought to be synonymous with gain (excitability) of post-synaptic pyramidal neurons reporting prediction errors (Parr, T. and K. J. Friston (2019). "Attention or salience?" Curr Opin Psychol **29**: 1–5.).

4 Technically, the reciprocal exchange between the inside (internal) and outside (external) across the Markov blanket means that the system is ‘open’, which calls on a very general formulation from physics in terms of nonequilibrium steady states Friston, K. (2019) "A free energy principle for a particular physics." arXiv:1906.10184.

5 Antibodies are divided into several classes depending upon their structure. These are (in order of prevalence) IgG, IgA, IgM, IgD, and IgE (where Ig means ‘immunoglobulin’). An individual antibody is made up of two structural components referred to as Fab (fragment antibody binding) and Fc (fragment crystallisable) regions.

6 C1q is broken down by C1-inhibitor. Absence of the latter underwrites the excessive activation of the complement pathway that characterises hereditary angioedema Busse and Christiansen (2020). "Hereditary Angioedema." New England Journal of Medicine **382**(12): 1136–1148.

7 Mast cells may also be triggered directly by the presence of a pathogen, via the IgE antibodies on their surfaces.

8 The killing of pathogens inside neutrophils (and macrophages) depends upon the NADPH oxidase enzyme. Congenital absence of this leads to an immunodeficiency called Chronic Granulomatous Disease (Arnold and Heimall 2017). "A Review of Chronic Granulomatous Disease." Advances in therapy **34**(12): 2543–2557. Granulomas are groups of phagocytic cells, normally macrophages, that have engulfed a pathogen but are unable to kill it. These also occur in conditions like tuberculosis (where the mycobacterium is resistant to oxidative killing) or sarcoidosis.

9 Empirical priors are Bayesian beliefs derived from higher levels of a hierarchical model. For example, a group mean may provide an empirical prior over an individual’s response to some treatment in classical (mixed effects) analyses of experimental data.

10 Given their central role in functioning of the adaptive immune system, it is unsurprising that certain HLA subtypes are associated with clusters of diseases with autoimmune features. Perhaps the most famous association is that of HLA-B27 with a group of disorders that includes psoriasis, ankylosing spondylitis, inflammatory bowel diseases, and reactive arthritis Bowness (2015). "HLA-B27." Annual Review of Immunology 33(1):29–48.

11 Immune cells specialising in defence against multicellular parasites and implicated in various hypersensitivities.

12 Technically, the internal and active states constitute *autonomous* states, in the sense that they do not depend upon external states. Crucially, one can always express their dynamics as a gradient flow on variational free energy. Heuristically, these gradients often have the form of prediction errors; namely, the difference between a sensory state and the prediction of that sensory state based upon the internal states. In other words, both internal and active states trying to minimise prediction errors; either by changing the internal milieu to adapt to sensory fluctuations or by acting upon the external milieu to realise predicted sensory states.

13 The ratio between the two types of T-helper cells (Th1 and Th2) is altered during pregnancy. A skew towards Th2 cells (which produce anti-inflammatory molecules) has been implicated in maintenance of healthy pregnancies, while a skew towards Th1 has been associated with recurrent miscarriages (Makhseed et al. 2001). "Th1 and Th2 cytokine profiles in recurrent aborters with successful pregnancy and with subsequent abortions." Hum Reprod **16**(10): 2219–2226.).

14 Under the free energy principle, a fundamental drive for me, as a self-organising system, is to maximise the accuracy of my predictions about the causes of my sensations, with models of the world that are as minimally complex as possible—in order to maximise evidence for my own existence. The human brain and immune system are two of the most complex systems in biology, which speaks to the evolutionary imperative for them to be impeccably accurate in their predictions.
